# Code Response Training: Improving Interprofessional Communication

**DOI:** 10.15766/mep_2374-8265.11155

**Published:** 2021-05-19

**Authors:** Heather Walsh, Laura Nicholson, Mary Patterson, Pavan Zaveri

**Affiliations:** 1 Simulation Program Manager, Simulation Program, Children's National; 2 Simulation Education Specialist, Simulation Program, Children's National; 3 Associate Dean and Professor, Lou Oberndorf Professor in Healthcare Technology, Center for Experiential Learning and Simulation, Department of Emergency Medicine, University of Florida; 4 Medical Director, Simulation Program, Division of Emergency Medicine, Children's National

**Keywords:** Simulation, Interprofessional Education, Communication Skills, Patient Safety, Quality Improvement, Pediatrics

## Abstract

**Introduction:**

Using simulation to improve team performance in emergencies is commonplace. Decreasing codes hospital-wide can be challenging. To address these needs, hospital leaders requested a simulation program to provide team training across an institution focused on patient safety and communication techniques.

**Methods:**

We developed a multimodal approach pairing three online modules on communication techniques with a simulation-based learning session. The three modules required 1 hour, followed by a 1-hour, in-person, simulation-based, interprofessional, small-group session of clinical staff. In ad hoc teams, participants managed two cases: a toddler with airway obstruction and a child developing septic shock. A focused debriefing included discussion of mental models, team formation and expertise, and communication techniques to create a common language to use in ad hoc team formation and patient care.

**Results:**

Through more than 200 training sessions reaching over 1,400 staff members, we executed code response training. A nurse and physician facilitated each session, emphasizing the interprofessional nature needed for patient care. Participants rated the learning experience highly on a 5-point Likert scale (1 = *low/poor*, 5 = *high/excellent*), with an average rating of 4.3 for achieving objectives and an average rating of 4.8 for facilitator effectiveness.

**Discussion:**

Through engaging leadership and frontline clinicians, the simulation program provided code response training hospital-wide, emphasizing the importance of teamwork and communication in critical situations. Such hospital-wide training can emphasize a shared language to empower clinicians at all levels to deliver safe, quality patient care.

## Educational Objectives

By the end of this activity, learners will be able to:

1. Demonstrate awareness of high-risk situations and errors leading to serious safety events in a pediatric hospital.
2. Apply team communication techniques known to decrease errors in a high-risk situation.
3. Demonstrate effective techniques for teamwork and communication in their daily work.

## Introduction

Simulation improves teamwork and technical skills.^1^ Dr. Hania Wehbe-Janek and colleagues advocate for the use of simulation training in preparing interprofessional teams for hospital emergencies, promoting effective teamwork and communication, and improving organizational culture.^2^ Related, transforming health care systems to high-reliability organizations has been advocated to minimize error in complex settings.^3^ The principles of high-reliability organizations are (1) preoccupation with failure, (2) reluctance to simplify, (3) sensitivity to operations, (4) commitment to resilience, and (5) deference to expertise.^4^ Bringing these principles into health care is critical to prevent harm to patients, yet the methods to accomplish this still elude health care organizations.^5^

Code response training was designed to address multiple objectives: team training, code response, and interprofessional education. Upon review of patient deteriorations, including multiple code blue events at our institution in acute care in 2015, it was determined that the lack of team communication in patient care delivery contributed to safety events. Since code blue events are rare, there are associated challenges, including the formation of ad hoc teams, the lack of confidence and competence with seldomly used skills, the lack of role clarity, and ineffective team communication. Communication techniques can ground clinicians when they are in high-risk situations, which helps them to prevent the loss of situational awareness and to achieve a shared mental model, ensuring a high level of team performance.

A previous simulation-based initiative focusing on teamwork and communication behaviors resulted in improved knowledge, safety attitudes, and changes in behavior during simulation.^6^ Based on a review of team performance measures, the most common communication techniques that we chose to focus on were assigning roles, identifying a leader, ensuring closed-loop communication, and sharing a mental model in ad hoc teams.^7–9^

Children's National was one of eight organizations awarded an ACGME Pursuing Excellence Pathway Innovators grant in 2016. In the ACGME's Executive Summary of the Clinical Learning Environment Review, one opportunity for growth identified for residents, fellows, and faculty members was experiential learning in patient safety.^10^ The aims of the Pursuing Excellence Pathway Innovators grant were to align strategic and graduate medical education strategies; incorporate quality, safety, equity, and value into the workflow; advance faculty expertise in quality and safety; and increase interprofessional learning opportunities.^11^ Code response training, which was the first initiative of the grant, focused on integrating communication techniques into clinical practice and expanding interprofessional learning within our organization through simulation. Hospital leaders sought the expertise of the Children's National simulation program to develop a program that reinforced desired communication techniques within our organization.

When reviewing prior trainings on patient safety, teamwork, and communication using simulation, we found several publications that focus on the physician trainee role regarding resuscitation training, such as Drs. Kevin Couloures and Christine Allen's training for pediatric residents^12^ and Dr. Ayesha Mirza and colleagues’ 4-hour training session for pediatric residents and fellows.^13^ Alternatively, in identifying interprofessional training, there are publications that focus on a specific setting such as the emergency department^14^ or the operating room.^15^ We also found interprofessional courses that have had limited numbers of participants, such as the course developed by Dr. Lisa Motz and colleagues,^16^ who focus on the medical resuscitation team members, and the course developed by Dr. Wei-Hsin Lu and collagues,^17^ who focus on internal medicine interns and senior nursing students. Our training focuses on the use of simulation to address patient safety through communication behaviors delivered across an entire hospital.

## Methods

### Development

In preparing this curriculum, the authors, a group of simulation educators, applied Kern's approach to curriculum development.^18^ The ACGME focus on interprofessional learning and addressing patient safety, the request of leadership at our institution to deliver team training, and the frequent code blue events at out institution formed the basis of the general and targeted needs assessment. After narrowing the focus to communication techniques related to patient safety, educational objectives were identified and a 2-hour curriculum—a combination of didactic and simulation-based learning—was designed as required training for all inpatient clinicians. The target audience included physicians, registered nurses, advanced practice providers, and respiratory therapists on all shifts across all inpatient areas and specialties. Implementation, further described below, was a significant challenge, as the target audience was about 1,400 inpatient clinicians. Evaluation focused on immediate and delayed feedback to determine retention of the techniques shared.

To reinforce team communication techniques and the principles of high-reliability organizations with busy clinicians, we determined that pairing online modules with an in-person simulation session was the best modality to meet the learning need. One of our physician simulationists, a renowned expert in patient safety, collaborated with our instructional designer to create three patient safety fundamentals online modules (Appendices A, B, and C), which were posted on an online quality improvement and safety portal. Within the portal, learners were able to complete the modules and print a certificate of module completion to bring to class. Instructions for viewing the online modules are included in Appendix D. Upon completion of the three online modules, clinicians registered for a 1-hour, interprofessional, simulation-based class held in the simulation center.

Each simulation training session was limited to four physicians or advanced practice providers, eight nurses, and two respiratory therapists. The 1-hour session consisted of two interprofessional simulations. All sessions were held in the simulation center to provide a uniform learning experience. Each session began with an introduction and briefing using a standard facilitator's guide (Appendix E). To ensure an effective team size for the simulation, we typically split the participants into two equal ad hoc teams, ensuring a mixture of nurses and physicians in each group. Both scenarios were designed to elicit communication between team members to deliver care to the patient. Appendices F and G, containing the simulation cases, and Appendix H, containing an equipment checklist for code response training, were provided to the groups. Participants were instructed that they would be the first responders to a call for help. The first group participated in a scenario involving a toddler in the hospital cafeteria with an occluded tracheostomy, in which the goal was to alleviate the plugged tracheostomy. Observers of the first case were asked to notice the use of communication techniques and comment as part of the debriefing using a checklist (Appendix I). The second group (observers of the first scenario) then participated in the second scenario, a 6-year-old child admitted on an acute care unit for osteomyelitis and now in septic shock. Peer feedback, particularly on the communication techniques, was incorporated into debriefings for both scenarios (Appendix J), reinforcing the concept of peer coaching.

### Equipment/Environment

Two high-fidelity simulators, one toddler and one pediatric, were used in the simulations. The scenarios were shared via cue cards, found in Appendices F and G, to indicate a state change of the manikin. The equipment checklist (Appendix H) included other equipment used to enhance fidelity of the scenarios. One scenario occurred in the cafeteria, requiring a table and two chairs with sheets covering up the walls to mimic a nonclinical area, while the other scenario occurred in an inpatient unit, requiring a bed and hospital room setup. Both simulations could be executed with low-fidelity manikins, as the focus was on the communication and teamwork of the participants.

### Personnel

Two to three facilitators, with at least one nurse and one physician, were required to facilitate each session, as well as a simulation technician to set up the simulations, run the manikins, and reset equipment between sessions. One facilitator played a family member, usually the patient's aunt or uncle, in each scenario. For both scenarios, the caregiver with the child would call out for help. For the inpatient scenario, one of the nurse participants was asked to play the role of the nurse covering for the patient. The covering nurse received a brief handoff (cue card) on the patient and could share that information with the first responders who were outside the room.

Facilitators for code response training included physician leaders in simulation, nurse simulation education specialists, and unit-based nurse educators and physicians who were engaged in patient safety work and simulation. Train-the-trainer sessions were held for facilitators to pilot the course and educate facilitators in the flow of the event prior to facilitating a course.

### Implementation

Simulation nurse and physician leaders attended key meetings of nursing and physician leaders to discuss this training and promote engagement. An email from the chief quality and safety officer was sent to physicians and advanced practice providers, while an email from the chief nursing officer was sent to nurses to complete this training requirement. Using an online system, we created sign-up slots for learners, with spaces for four physicians/advanced practice providers, up to eight nurses, and two respiratory therapists for each 1-hour session.

Sessions were scheduled during weekday business hours over a 4-month period. Night and weekend sessions were discussed but not offered due to historical poor attendance during those times. The simulation team maintained an attendance spreadsheet from the paper sign-in sheets that was periodically sent to leadership at the unit/division level for compliance. Upon completion of the online and live components of code response training, nurses received 2 contact hours. Continuing medical education (CME) was discussed, but this was believed to be less of an incentive for physicians, as our institution offered many opportunities for physicians to obtain CME.

### Evaluation

This initiative had a robust evaluation plan with follow-up surveys at identified time points. Participant evaluations were obtained at the end of each session (Appendix K). Participants evaluated their ability to achieve each of the learning objectives, the effectiveness of the RN and MD facilitators, and the application and usefulness of the content. Additionally, participants identified new or reinforced communication techniques in this simulation event and which technique they planned to apply to their own and their team's practice. A short-term evaluation occurred 4 to 6 weeks postcourse in which we asked learners about the techniques they had applied over the past months and the barriers and enablers to using these techniques in practice (Appendix L). Lastly, participants were sent a long-term follow-up activity 4 to 6 months postcourse that included matching and true/false questions about communication techniques and reflections on takeaways and supports for applying those techniques and takeaways to practice (Appendix M). This was a requirement for nurses to obtain continuing nursing education (CNE) credit. Upon completion of both follow-up activities, nurses were able to download and print the certificate of completion for CNE from the online portal.

### Debriefing

Since we needed to thoroughly orient learners to each session and run two scenarios in 1 hour, each debriefing was focused and concise. Typically, each scenario lasted 5–7 minutes, followed by a debriefing of 15–20 minutes. To ensure debriefings included discussion of certain points and to maintain consistency, we created a debriefing guide (Appendix J) along with sample responses to direct the discussion.

Debriefing focused on eliciting the mental model from participants, if not stated during the case; formation of the ad hoc team and determining expertise; escalating care; and reinforcing useful communication techniques, including techniques used and/or not used by participants. Secondary areas of discussion for the first scenario included what escalation of care means and availability of supplies in a non-patient care area such as the cafeteria. For the second scenario, other areas of discussion included contrasting a rapid response call versus code blue, expectations on ordering fluids, use of the push-pull method for boluses, and medications available on acute care units.

## Results

We held 230 sessions from October 2016 to February 2017, with 1,408 clinicians participating in the code response training: 801 nurses, 419 physicians, and 188 other clinicians (including advanced practice providers and respiratory therapists). Of participants, 57% were nurses. Participants included day and night shift staff who were able to come in on a day off or attend an early morning class. Since this was required education and the in-person class was only 1 hour, managers and directors supported their staff attending, and some nurses were able to cover assignments so that they could attend a session during a clinical shift.

On the immediate postcourse evaluation, participants rated achievement of the three educational objectives and the effectiveness of the facilitators based on a 5-point Likert scale (1 = *low/poor*, 5 = *high/excellent*). Participants rated achievement of both the first educational objective (demonstrate awareness of high-risk situations and errors leading to serious safety events in a pediatric hospital) and second educational objective (apply team communication techniques known to decrease errors in a high-risk situation) at an average of 4.3. Participants rated achievement of the third objective (demonstrate effective techniques for teamwork and communication in their daily work) at an average of 4.4. Participants rated the RN and MD facilitators’ effectiveness at an average of 4.8. Of participants, 88% indicated that the session provided information that would change their practice. When asked about what techniques were new or reinforced in this simulation event, the following themes emerged:

• Establishing a team leader.
• Sharing a mental model.
• Using closed-loop communication.
• Identifying roles and responsibilities in a critical situation.
• Escalating care.
• Increased awareness of hospital resources/procedures.
• Thinking out loud to enhance situational awareness.

Of participants, 96% reported the physical facilities were conducive to learning. In additional comments, participants offered that the module content was much needed and well developed. Several learners had difficulty with the audio and felt the pace of the modules was slower than necessary.

The evaluation sent 4 to 6 weeks following code response training sought to elicit which techniques learners had integrated into practice along with barriers and enablers to integration. On analyzing the responses, the predominant themes were accessing resources, team functioning, and communication. Representative quotes from the learners for techniques applied included:

• “We had a code in the cafeteria, and I think I was able to communicate better with the other responders.”
• “Closed-loop communication between myself and other health care professionals. Also, the importance of expressing concern and verbalizing what I may be thinking inside, without being scared of being wrong.”

By contrast, in explaining barriers to integrating techniques into practice, learners stated:

• “At first, my confidence was a barrier because I did not want to necessarily speak up for fear of being wrong; however, after the simulation I learned that we are a team here to better the patient and it will be okay to speak up—worst case scenario it is an educational moment.”
• “As a new grad nurse, I have trouble speaking up because I am not confident in myself yet. I am afraid of saying the wrong thing.”
• “As the RN, you don't always feel like the MDs listen to you. Being confident in my expertise and knowing when to speak up when I have a gut feeling.”
• “Potentially openness to these skills/forms of communication for medical professionals who have not practiced these techniques—high stress situations that often result in reverting to old habits.”

Additionally, learners related the following enablers to practicing communication techniques:

• “Having interdisciplinary training, which reinforced the importance of having closed-loop communication and a shared mental model across the organization.”
• “I think it helped that so many people participated in the code response simulation because it really forced people to look at the situation from other people's perspectives, and has helped create an environment where everyone feels comfortable voicing their concerns.”

The final evaluation, which occurred 4 to 6 months posttraining, included matching, true/false, and behavior reflection questions intended to capture practice changes. Of participants, 425 out of 1,408 responded, a 30% response rate. Most of the responses were by RNs, as completing the final evaluation was a required component to obtain CNE for the curriculum. Of the four matching questions, 67% had all correct answers. For the true/false questions, 96% of learners were correct on question 2 (standardized communication), 55% on question 3 (situational awareness), and 60% on question 4 (assertiveness). For question 5 (takeaways), the key communication techniques were noted at the following frequencies: assigning roles (*n* = 50), identifying a leader (*n* = 10), ensuring closed-loop communication (*n* = 126), and sharing a mental model (*n* = 34). For question 6 (what else was needed?), the most frequent comments were none, N/A, or nothing (*n* = 235). From the remaining 190 responses, the most frequent comments were practice (*n* = 93), communication (*n* = 44), and simulation (*n* = 43).

True code blue events in acute care in our organization occurred frequently in 2015, with a total of 18 in that calendar year. There was a 72% decrease from 2015 to 2016, with five events in 2016. These events continue to remain low in frequency, with seven code blue events in acute care in 2017 and five in 2018, which we in part attribute to code response training. Other parallel efforts included interprofessional in situ simulation training with nurses and residents on escalation of care, unit-based teams focused on decreasing late rescue events, and a focus on the culture of safety by patient safety and hospital leadership.

## Discussion

Code response training, the largest simulation-based training at our institution, describes an interprofessional, hospital-wide, simulation-based initiative aimed at improving safety culture. Through the code response training program, the simulation program demonstrated the efficacy of hospital-wide teamwork training and emphasized the importance of communication and teamwork in critical situations. The online and simulation-based components of this initiative can be easily adapted and used in various health care settings.

The scenarios were intentionally crafted to be feasible for ad hoc teams from throughout the institution. Pilot testing of the scenarios provided an opportunity to integrate specific details into the case to increase realism (e.g., family member with tracheostomy bag in the cafeteria). An emergency scenario in the hospital cafeteria proved to be thought provoking for most teams, since it is uncommon and staff were generally unaware of which supplies, if any, were available. It led to discussion about the importance of calling a code blue in non-patient care areas, how to call a code, code team responders, and the equipment that is brought in as part of a code blue activation. Managing tracheostomies was not familiar to all clinicians. Certain specialists, such as ear, nose, and throat specialists and physical medicine and rehabilitation specialists, were experts in managing tracheostomies, while others (e.g., psychiatrists) were not. Interestingly, psychologists and other specialists found it more natural to speak with the aunt to obtain pertinent patient information, provide information, and comfort her. In a particularly memorable session, a nurse repeatedly picked up the emergency tracheostomy tube to suggest its utility without saying a word. This was explored in the debriefing, and the nurse explained that she was waiting for the physician to direct the care. The physician, an orthopedic surgeon, admitted to not knowing about tracheostomies. These actions demonstrated in the scenarios led to debriefing about deference to expertise and the importance of introductions with a brief one-line statement about one's role and expertise/experience, particularly when in an ad hoc team. The openness of staff as learners in the simulations and the use of first names in the training helped to flatten the organizational hierarchy.

The septic shock scenario was problematic for participants, in that the responders were passersby in the hallway headed to buy a snack and were not responding to one of their patients. One caveat was that some of the critical care clinicians were preparing to perform intensive care procedures on an acute care unit with staff who were uncomfortable with the situation. There were some misunderstandings of how to call for help on acute care units versus critical care units and incorrect assumptions, even from experienced clinicians. A final challenge stemmed from specialization. In one team, a cardiologist suspected supraventricular tachycardia (our septic patient was tachycardic), while a neurologist suspected stroke (lethargy). This highlighted the need for sharing a mental model to establish the team is on the same page in order to proceed with treatment.

Because messaging about this course from leadership was brief, we began with a detailed explanation of why the course was being implemented in addition to setting the stage for the safe learning environment that is typical in simulation. This was critical for faculty participating in the course as this was their first experience with simulation. For the nurses, many said it was their first time learning with attending physicians. Given the large number of sessions held, it was important to keep the message consistent across classes and facilitators, which was achieved using the facilitator's guide for code response training (Appendix E). Providing this education to hundreds of clinicians across all inpatient units, including acute and critical care and various departments and specialties, created a foundation on which staff felt empowered to use the communication techniques in real patient care situations. Participants were learners in the sessions, despite their positions, leading to a unique opportunity for reflection and discussion in the debriefings about deference to expertise and hierarchy and how the teams could be maximized to help the patient in each case. Since we offered hundreds of sessions, we purposely included a statement in the orientation asking that participants not share the specifics of either scenario to ensure learning occurred in all sessions. We felt that this request was upheld, as learners in the last sessions did not seem to have prior knowledge of either case.

There was much positive feedback from clinicians following the course, including from faculty who encouraged nurses, who typically have experience with tracheostomies, to speak up and lead, and from nurses who felt empowered to lead with provider encouragement.

Lessons learned from this training include scheduling fewer sessions each day, either morning or afternoon, to avoid facilitator fatigue. Offering sessions to all clinical staff allowed us to focus on critical behaviors for ad hoc team formation, while the disadvantage was staff not learning within their native teams. Simulation performance in this curriculum demonstrated the need to design future interventions focusing on establishing leadership, identifying roles and responsibilities, and other areas of improvement. Next steps include identifying opportunities to focus on these areas within in situ simulations and assessing the effectiveness of these interventions. In situ simulations provide a method to reinforce teamwork behaviors in the clinical setting, and the use of these techniques (e.g., creation of a shared mental model, summary statements, and role clarity) can be acknowledged and debriefed.^19^

Limitations of this training included a small simulation team and simulation center. Other limitations included a limited number of classes offered, focus on inpatient providers only, and staff turnover, limiting our ability to capture all staff. Technical challenges were glitches encountered when launching a new portal and limited availability of audio at computers where clinicians worked in patient care areas to complete the modules during a quiet period. There was a lack of incentive for physicians to complete the subsequent evaluations without continuing education credit. Lastly, there were limitations in evaluation response, including incomplete follow-up and limited responses, particularly to the evaluation 4 to 6 months postcourse.

Implementation required engagement of hospital leaders, unit-based educators, nursing directors, and division chiefs. The online learning platform enabled content delivery, data collection, evaluation management, and further iterative course development. Online modules provided foundational knowledge for all clinicians, while the simulation-based training provided the ideal forum to allow interprofessional ad hoc teams an opportunity to practice using the techniques in a safe space. The debriefing encouraged teams to ask questions, reflect on what went well, and identify opportunities for improvement in future situations. The overwhelmingly positive response to this initiative from frontline clinicians and hospital leadership—even our surgeon CEO attended a session—garnered support for continued hospital-wide, interprofessional, simulation-based training. Training throughout an institution is a formidable task, but our experience shows it is feasible with engagement, leadership, and broadly applicable material.

## Appendices

Module 1 Patient Safety Fundamentals folderModule 2 Communication and Teamwork folderModule 3 Pulling It Together folderModule Instructions.docxFacilitators Guide.docxSimulation Case 1.docxSimulation Case 2.docxEquipment Checklist.docxObserver Checklist.docxDebriefing Guide.docxPostcourse Evaluation.docxShort-Term Follow-Up Activity.docxLong-Term Follow-Up Activity.docx
*All appendices are peer reviewed as integral parts of the Original Publication.*

## References

[1] Patterson MD, Geis GL, LeMaster T, Wears RL. Impact of multidisciplinary simulation-based training on patient safety in a paediatric emergency department. BMJ Qual Saf. 2013;22(5):383–393. 10.1136/bmjqs-2012-000951 23258388

[2] Wehbe-Janek H, Lenzmeier CR, Ogden PE, et al. Nurses’ perceptions of simulation-based interprofessional training program for rapid response and code blue events. J Nurs Care Qual. 2012;27(1):43–50. 10.1097/NCQ.0b013e3182303c95 21849908

[3] Baker DP, Day R, Salas E. Teamwork as an essential component of high-reliability organizations. Health Serv Res. 2006;41(4)(pt 2):1576–1598. 10.1111/j.1475-6773.2006.00566.x 16898980 PMC1955345

[4] Weick KE, Sutcliffe KM. Managing the Unexpected: Sustained Performance in a Complex World. 3rd ed. John Wiley and Sons; 2015.

[5] Cochrane BS, Hagins M Jr, Picciano G, et al. High reliability in healthcare: creating the culture and mindset for patient safety. Healthc Manage Forum. 2017;30(2):61–68. 10.1177/0840470416689314 28929881

[6] Patterson MD, Geis GL, Falcone RA, LeMaster T, Wears RL. In situ simulation: detection of safety threats and teamwork training in a high risk emergency department. BMJ Qual Saf. 2013;22(6):468–477. 10.1136/bmjqs-2012-000942 23258390

[7] Reid J, Stone K, Brown J, et al. The Simulation Team Assessment Tool (STAT): development, reliability and validation. Resuscitation. 2012;83(7):879–886. 10.1016/j.resuscitation.2011.12.012 22198422

[8] Malec JF, Torsher LC, Dunn WF, et al. The Mayo High Performance Teamwork Scale: reliability and validity for evaluating key crew resource management skills. Simul Healthc. 2007;2(1):4–10. 10.1097/SIH.0b013e31802b68ee 19088602

[9] Grant EC, Grant VJ, Bhanji F, Duff JP, Cheng A, Lockyer JM. The development and assessment of an evaluation tool for pediatric resident competence in leading simulated pediatric resuscitations. Resuscitation. 2012;83(7):887–893. 10.1016/j.resuscitation.2012.01.015 22286047

[10] Wagner R, Patow C, Newton R, Baretta RC, Koh NJ, Weiss KB; CLER Program. The overview of the CLER Program: CLER National Report of Findings 2016. J Grad Med Educ. 2016;8(2)(suppl 1):11–13. 10.4300/1949-8349.8.2s1.11 27252798 PMC4888445

[11] Bagian JP, Weiss KB; CLER Evaluation Committee. The overarching themes from the CLER National Report of Findings 2016. J Grad Med Educ. 2016;8(2)(suppl 1):21–23. 10.4300/1949-8349.8.2s1.21 27252800 PMC4888453

[12] Couloures KG, Allen C. Use of simulation to improve cardiopulmonary resuscitation performance and code team communication for pediatric residents. MedEdPORTAL. 2017;13:10555. 10.15766/mep_2374-8265.10555 30800757 PMC6342167

[13] Mirza A, Winer J, Garber M, Makker K, Maraqa N, Alissa R. Primer in patient safety concepts: simulation case-based training for pediatric residents and fellows. MedEdPORTAL. 2018;14:10711. 10.15766/mep_2374-8265.10711 30800911 PMC6342435

[14] Wong A, Gang M, Wing L, Ng G, Szyld D, Mahoney H. Team training for success: an interprofessional curriculum for the resuscitation of emergency and critical patients. MedEdPORTAL. 2014;10:9807. 10.15766/mep_2374-8265.9807

[15] Wongsirimeteekul P, Mai CL, Petrusa E, et al. Identifying and managing intraoperative arrhythmia: a multidisciplinary operating room team simulation case. MedEdPORTAL. 2018;14:10688. 10.15766/mep_2374-8265.10688 30800888 PMC6342395

[16] Motz L, Lloyd B, Donato A, et al. Interdisciplinary curriculum and simulation cases for teaching leadership and communication to medical rapid response teams. MedEdPORTAL. 2012;8:9145. 10.15766/mep_2374-8265.9145

[17] Lu WH, Goolsarran N, Hamo CE, Frawley SM, Rowe C, Lane S. Teaching patient safety using an interprofessional team-based learning simulation model in residency training. MedEdPORTAL. 2016;12:10409. 10.15766/mep_2374-8265.10409 31008189 PMC6464414

[18] Thomas PA, Kern DE, Hughes MT, Chen BY, eds. Curriculum Development for Medical Education: A Six-Step Approach. 3rd ed. Johns Hopkins University Press; 2016.

[19] Wheeler DS, Geis G, Mack EH, LeMaster T, Patterson MD. High-reliability emergency response teams in the hospital: improving quality and safety using in situ simulation training. BMJ Qual Saf. 2013;22(6):507–514. 10.1136/bmjqs-2012-000931 23457361

