# Supplementary material for: Code Response Training: Improving Interprofessional Communication
Source: MedEdPORTAL. 2021 May 19;17:11155. doi: 10.15766/mep_2374-8265.11155 (PMC8131416; doi:10.15766/mep_2374-8265.11155)
Supplement: Supplementary file 1 — Module 1 Patient Safety Fundamentals folderModule 2 Communication and Teamwork folderModule 3 Pulling It Together folderModule Instructions.docxFacilitators Guide.docxSimulation Case 1.docxSimulation Case 2.docxEquipment Checklist.docxObserver Checklist.docxDebriefing Guide.docxPostcourse Evaluation.docxShort-Term Follow-Up Activity.docxLong-Term Follow-Up Activity.docx [file mep_2374-8265.11155-s001.zip › B. Module 2 Communication and Teamwork/index.html]

Communication and Teamwork


Communication and Teamwork
