# Supplementary material for: Code Response Training: Improving Interprofessional Communication
Source: MedEdPORTAL. 2021 May 19;17:11155. doi: 10.15766/mep_2374-8265.11155 (PMC8131416; doi:10.15766/mep_2374-8265.11155)
Supplement: Supplementary file 1 — Module 1 Patient Safety Fundamentals folderModule 2 Communication and Teamwork folderModule 3 Pulling It Together folderModule Instructions.docxFacilitators Guide.docxSimulation Case 1.docxSimulation Case 2.docxEquipment Checklist.docxObserver Checklist.docxDebriefing Guide.docxPostcourse Evaluation.docxShort-Term Follow-Up Activity.docxLong-Term Follow-Up Activity.docx [file mep_2374-8265.11155-s001.zip › D. Module Instructions.docx]

**Appendix D. Module Instructions**

These modules will best function when placed in a learning management system (LMS). This will allow the facilitators to track usage, completion and deliver the follow-up activities.

To view the modules:

1. First download the publication .zip file from *MedEdPORTAL*.
2. Right-click on the publication .zip folder and click Extract all from the Zipped File (this unzipping will happen automatically on an Apple/Mac).
3. In the new publication folder where the files were extracted, you will see three sub folders for each of the modules.
4. Click on index.html file within the folder, which will open the module in your web browser. You can then navigate the full module from the web browser.
