# Supplementary material for: Code Response Training: Improving Interprofessional Communication
Source: MedEdPORTAL. 2021 May 19;17:11155. doi: 10.15766/mep_2374-8265.11155 (PMC8131416; doi:10.15766/mep_2374-8265.11155)
Supplement: Supplementary file 1 — Module 1 Patient Safety Fundamentals folderModule 2 Communication and Teamwork folderModule 3 Pulling It Together folderModule Instructions.docxFacilitators Guide.docxSimulation Case 1.docxSimulation Case 2.docxEquipment Checklist.docxObserver Checklist.docxDebriefing Guide.docxPostcourse Evaluation.docxShort-Term Follow-Up Activity.docxLong-Term Follow-Up Activity.docx [file mep_2374-8265.11155-s001.zip › E. Facilitators Guide.docx]

Code Response Training Simulation Facilitator’s Guide

Thank you for attending today. We are glad you are here. In our efforts to continue to be a top hospital in the country, we believe that everyone wants to take the best possible care of our patients. The modules and these simulations aim to get all of us there.

- The simulations today are meant to be formative-to help us improve not as a “test”. We are focusing on non-technical skills to improve our communication and teamwork.
- We are interested in the team’s performance. We don’t care if a single individual (point to someone or use a name) knows the dose of epinephrine, we do care that you as a team know how to use your resources and find the necessary information.

- The simulations are meant to be challenging. If they are not challenging, we are wasting your time. We don’t expect you to perform perfectly. We expect you to make mistakes.
- Since that is the case, we operate under “Vegas Rules”. What happens in simulation, stays in simulation. We promise you that we will not discuss any individual’s performance outside of the simulation lab. No individual’s performance will be discussed with a supervisor, director, or anyone else. We also expect that all simulation participants will not discuss any other participant’s simulation performance outside the lab.
- We understand that the manikins are not real patients; the environment is not your actual clinical environment and that you may not act as you normally act in a clinical situation. Having said all that, we ask you to engage in these simulations as real clinical situations and that you treat the patients as real and do what you would actually do in a clinical situation. Like Mr. Rogers Neighborhood and the Land of Make Believe- even though it is not real, you can still learn something here.
- Additionally, while we encourage you to share the learning from the simulation with your colleagues, we ask that you not discuss specific scenarios. A tremendous amount of effort goes into developing scenarios and sharing the specifics of a scenario robs others of their opportunity to learn from the scenario. Besides, you wouldn’t want anyone to do better than you did. ☺
- We will orient you to the simulator and the environment. This is not a “mock code.” If you want to know if the patient is wheezing, you need to listen. If you want to know if the patient has a pulse, you need to check the patient’s pulse. Simulation is a contact sport and everyone on the team will be involved.
- Again, recall that we are focusing on non-technical skills. As you reviewed in the modules, we would expect to hear you using shared mental models, closed loop communications, clarifying questions, etc.
- Have fun!

The Simulation Center and Manikins

- The manikins we will use today have radial and carotid pulses.
- They can breathe, have chest rise and can talk.
- They can become cyanotic when their hypoxia is severe.
- IV fluids and medications can be given via the access provided and/or any access you obtain and need to be actually given as you would in a real event.
- Supplies will be provided as appropriate for the environment and clinical situation.
- If a code cart is accessed, keep in mind that a Code Book is available on the top with a variety of resources including and weight-specific medication dosing and equipment sizes.

**Orient to Scenario 1**

- You are in the cafeteria getting your lunch with many others as usual.
- A parent calls out to you.

**Orient to Scenario 2**

- You are on the inpatient unit getting a snack at the snack bar, doing consults or providing bedside care as you normally would.
- The patient will be a hospitalized patient, so there is a code sheet of medications on the bedside table for you.
- If you are going to give medications or fluids, you will attach that to the blue hub and actually give them.
- There is no oxygen behind the headwall. No oxygen will actually flow. Perform all actions as you normally would.
- The monitor at the bedside is connected with heart rate, pulse ox and respiratory rate available in that order. If you need a blood pressure, you must place the cuff on the patient and a blood pressure will be measured.
- A thermometer is available at the bedside and can be used to measure the temperature which will show on the monitor in the bottom right corner.
- Questions?
- Let’s get started.
- You will be the covering nurse for the bedside nurse who is at Code Response Training with limited sign-out information regarding this patient.
