# Supplementary material for: Code Response Training: Improving Interprofessional Communication
Source: MedEdPORTAL. 2021 May 19;17:11155. doi: 10.15766/mep_2374-8265.11155 (PMC8131416; doi:10.15766/mep_2374-8265.11155)
Supplement: Supplementary file 1 — Module 1 Patient Safety Fundamentals folderModule 2 Communication and Teamwork folderModule 3 Pulling It Together folderModule Instructions.docxFacilitators Guide.docxSimulation Case 1.docxSimulation Case 2.docxEquipment Checklist.docxObserver Checklist.docxDebriefing Guide.docxPostcourse Evaluation.docxShort-Term Follow-Up Activity.docxLong-Term Follow-Up Activity.docx [file mep_2374-8265.11155-s001.zip › F. Simulation Case 1.docx]

| **Appendix F: MedEdPORTAL Simulation Case**  **SIMULATION CASE TITLE:** Code Response Training Simulation #1- Toddler with Occluded Tracheostomy in the Cafeteria  **AUTHORS:** Heather Walsh MSN RN PCNS-BC CHSE CPN, Laura Nicholson MSN RN CHSE CPN, Mary Patterson MD MEd, Pavan Zaveri MD MEd CHSE  **LEARNER AUDIENCE**: Inpatient clinicians (physicians, fellows, nurses, advanced practice providers, respiratory therapists) | |
| --- | --- |
| **PATIENT NAME:** Timmy  **PATIENT AGE:** 18 months  **CHIEF COMPLAINT:** 18 mo M with trach, has been doing well, at hospital for clinic visit and noted in cafeteria to be having trouble breathing and turning blue. Require trach change for resolution as trach is plugged with mucus. Was eating grapes just before this happened to have team consider choking.  **PHYSICAL SETTING:** Cafeteria (performed in Simulation Center with curtains hung to cover physiologic monitor and patient headwall) with table and chairs; only medical equipment available is the trach bag with the aunt accompanying the patient | |
|  | |
| **Brief narrative description of case** | 18 mo M in cafeteria with aunt who suddenly develops respiratory distress while eating. The patient is initially fussy and experiencing respiratory distress (retractions, nasal flaring, perioral cyanosis) with diminished breath sounds bilaterally. The cause is an occluded tracheostomy; resolution requires identification of the problem, asking the aunt for the emergency trach bag, and changing the trach. Failure to correctly identify and treat the occluded trach leads to a worsening respiratory exam with poor air movement and chest rise, decreased respiratory effort, and lethargy.  Anticipated team actions include formation of ad hoc team in initial management, escalation of care and demonstration of safety communication techniques. |
| **Primary Learning Objectives** | By the end of this simulation, the learner will be able to:  •Assess a deteriorating patient using a structured approach (ABCDE, SAMPLE hx)  •Include technology-related emergency in diagnostic work up of respiratory distress  •Create a management plan for tracheostomy occlusion  •Use communication strategies to ensure safety in high risk situations (SBAR, closed loop communication) |
| **Critical Actions** | Assessment:   - ABCDE assessment - Brief SAMPLE history - Recognize respiratory distress/ cyanosis   Actions:   - Expose patient - Call Code Blue - Request someone to get the AED - Seek information from aunt; ask for emergency trach bag - Change trach, as no other equipment available in the cafeteria   Clinical Reasoning:   - Develop differential diagnosis of respiratory distress in tracheostomy dependent child   Teamwork/Communication:   - Introductions as team convenes with role identification (e.g., “I’m Joe, PICU fellow. I can be the leader”, or I’m Emily, a nurse and have experience with trach patients”). - Shared mental model - Thinking out loud - Closed loop communication (e.g., “someone call a code”; “I’ve just called a code blue”). |
| **Learner Preparation or Prework** | All learners had completed 3 online Patient Safety Fundamentals modules prior to the simulation-based session. |

| Initial Presentation | |
| --- | --- |
| **Initial vital signs** | Since this case occurred in the cafeteria, the responders do not have access to monitors. A cue card was placed after initial assessment with information on respiratory effort and trach assessment. |
| **Overall Setting and Appearance** | The location is intended to be the cafeteria. We used a bedside table to mimic a cafeteria table with 2 chairs around the table. The manikin has a tracheostomy in place and is dressed in shorts and a shirt. There is an open bag of grapes on the table. The aunt has an emergency trach bag with her, but provides it when asked. |
| **Confederates (e.g., standardized participants) and their roles in the room at case start** | One of our facilitators played the role of the aunt/uncle.  Caregiver information:  You are the aunt/uncle in the cafeteria with your 18 month-old nephew who suddenly develops respiratory distress while eating. He has a trach, but you don’t know why. Initially, asking someone to help him. Then “he’s having trouble breathing and turning blue”. If team fails to act, “he doesn’t look like he’s breathing; please help him”.  He’s been doing well; the trach is supposed to be coming out soon. Your sister ran to the pharmacy to get some prescriptions while waiting for Timmy’s appointment.  (provide only when asked): Tommy had problems early on in life requiring trach, but has been doing well since with normal behavior, activity, and development  If asked, you have a (emergency trach) bag your sister left with you, but aren’t sure what’s in there or what to do. |
| **HPI** | Initial patient information provided to participants:  18 mo M in cafeteria with aunt who suddenly develops respiratory distress while eating.  Significant Lab Values (provide only when asked): None  Significant Study Results (provide only when asked): None  Additional Information (provide only when asked): Had problems early on in life requiring trach, but has been doing well since with normal behavior, activity, and development  Recent Events: None  Allergies: NKDA |
| **Physical Examination** | |
| **General** | Very fussy and in distress  Perioral cyanosis |
| **HEENT** | Nasal flaring |
| **Neck** | Tracheostomy tube in place without redness or drainage |
| **Lungs** | Diminished breath sounds bilaterally without wheeze or rales  Diffuse retractions |
| **Cardiovascular** | Tachycardia (if auscultated) |
| **Abdomen** | Normal exam |
| **Neurological** | Normal exam |
| **Skin** | Normal |
| **GU** | Normal exam |

| Instructor Notes - Changes and CASE Branch Points | | |
| --- | --- | --- |
| **Intervention / Time point** | **Change in Case** | **Additional Information** |
| Initial assessment | Very fussy and in distress  Perioral cyanosis  Diffuse retractions  Nasal flaring  Diminished breath sounds bilaterally without wheeze or rales  Tracheostomy tube in place without redness or drainage | *Aunt: “Help him. He’s having trouble breathing. What’s going on? He seems to be getting worse. He’s turning blue”.*  Team should assess patient and seek information from aunt. Team should recognize respiratory distress and need for help. |
| Team may activate a code blue from the cafeteria (verbalize calling and the correct number to call).  There is no medical equipment present, except staff with stethoscopes. | No change | Code cart from central supply brought by a technician will arrive a few minutes later. However, code cart does not contain suction, oxygen, or trachs. The Code team arrives from various other locations in the hospital. |
| Repeat assessment | Lethargic  Decreased respiratory effort  Facial cyanosis  Poor air movement  Poor chest rise | *Aunt: “He doesn’t look like he’s breathing. I don’t see his chest moving. Timmy! Timmy! Should I call my sister? Is he going to die”?*  Expect worsening respiratory status to prompt identification of need for trach change and request for trach bag from aunt. |
| If staff asks for trach bag, aunt will supply it. If team chooses, may change trach in cafeteria. | Upon successful trach change, Timmy begins coughing, breathing, cyanosis resolves. | *Aunt: “You saved Timmy. Thank you so much. He seems so much better”.* |
| CPR initiated / No action taken | Facilitator ends case if tracheostomy occlusion not addressed to move to debriefing. | Occasionally, the team misinterpreted the cyanosis for cardiopulmonary arrest. |

**Ideal Scenario Flow**

Clinicians walking through the cafeteria hear a woman yelling for help and find her sitting with a toddler who is in obvious respiratory distress with a bag of open grapes on the table. They immediately designate someone to call a code blue, either from the cafeteria wall phone or a cell phone. Team members would be expected to perform brief introductions as they assess the child, appreciating his respiratory distress and the lack of available equipment. The team speaks with the woman, who is the boy’s aunt, to gather additional information about Timmy. We would expect to hear a shared mental model; ideally trach plug/ occlusion. Clinicians may auscultate breath sounds, if they have a stethoscope and may also move the child onto the table or the floor. Without intervention, the providers note that the patient’s respiratory status has continued to worsen. If asked, the aunt may provide the emergency trach bag, at which point, we would expect the team to identify the correct trach and perform a trach change. At this point, the patient will begin coughing and respiratory status will quickly improve.

**Anticipated Management Mistakes**

1. Difficulty with calling a code: We found that many nurses knew the number to call a code blue, but many faculty did not. Additionally, in our institution calling a code blue differs when calling from an in-house phone (4 digits) vs. a personal cell phone. There is not a code blue button in the cafeteria, as there are in patient care areas. Some clinicians wanted to call a rapid response, which is designated only for inpatient acute care areas and includes a 15-minute response. These misconceptions were addressed in the debriefing, as it is critical to be able to call for help in an emergency.
2. Failure to recognize the need for trach change: Some of our clinicians did not immediately recognize that the patient had an occluded or plugged trach, leading to delay in treatment. Others believed the patient was choking or experiencing anaphylaxis, leading to incorrect interventions. Occasionally, the team did not intervene or started CPR misinterpreting the cyanosis to indicate cardiorespiratory arrest. The worsening respiratory status and the aunt’s behavior escalating prompted the need for immediate intervention. Some staff who recognized the need to change the trach admitted to being hesitant to do so in a common area like the cafeteria. The debriefing focused on the importance of sharing the mental model (trach occlusion vs. choking) to ensure the team can proceed with interventions to help the patient, deference to expertise (trach experience critical in this scenario), and seeking information from the parents/caregivers.
3. Uncertainty about available equipment in non-clinical setting: Many of our learners were unfamiliar with the medical equipment available in the cafeteria (none), location of nearest AED to the cafeteria, and on the code cart. For example, portable suction, oxygen, and trachs are not part of the code cart. A few learners were unfamiliar that trach patients need to carry equipment with them in case of emergencies. We covered these equipment questions in the debriefing.

Cue Card Information

Physical Exam:

Very fussy and in distress

Perioral cyanosis

Diffuse retractions

Nasal flaring

Diminished breath sounds bilaterally without wheeze or rales

Tracheostomy tube in place without redness or drainage

Repeat Exam:

Lethargic

Decreased respiratory effort

Facial cyanosis

Poor air movement

Poor chest rise
