# Supplementary material for: Code Response Training: Improving Interprofessional Communication
Source: MedEdPORTAL. 2021 May 19;17:11155. doi: 10.15766/mep_2374-8265.11155 (PMC8131416; doi:10.15766/mep_2374-8265.11155)
Supplement: Supplementary file 1 — Module 1 Patient Safety Fundamentals folderModule 2 Communication and Teamwork folderModule 3 Pulling It Together folderModule Instructions.docxFacilitators Guide.docxSimulation Case 1.docxSimulation Case 2.docxEquipment Checklist.docxObserver Checklist.docxDebriefing Guide.docxPostcourse Evaluation.docxShort-Term Follow-Up Activity.docxLong-Term Follow-Up Activity.docx [file mep_2374-8265.11155-s001.zip › G. Simulation Case 2.docx]

| **Appendix G: MedEdPORTAL Simulation Case**  **SIMULATION CASE TITLE:** Code Response Training Simulation #2- Inpatient in Septic Shock  **AUTHORS:** Heather Walsh MSN RN PCNS-BC CHSE CPN, Laura Nicholson MSN RN CHSE CPN, Mary Patterson MD MEd, Pavan Zaveri MD MEd CHSE  **LEARNER AUDIENCE**: Inpatient clinicians (physicians, fellows, nurses, advanced practice providers, respiratory therapists) | |
| --- | --- |
| **PATIENT NAME:** Johnny  **PATIENT AGE:** 6 years  **CHIEF COMPLAINT:** 6 year-old boy admitted 5 days ago for an extensive osteomyelitis and abscess and is here receiving IV antibiotics. He is being treated with IV Clindamycin through his PICC line. He was noted to be tachycardic to 130 earlier with fever and started on IV fluids for suspected dehydration. He requires fluid resuscitation and consideration of pressors.  **PHYSICAL SETTING:** Inpatient acute care unit; patient in bed on monitor with mother at bedside | |
|  | |
| **Brief narrative description of case** | Patient is a 6 year-old admitted 5 days ago for an extensive osteomyelitis and abscess and is here receiving IV antibiotics. He is being treated with IV Clindamycin through his PICC line. He was noted to be tachycardic to 130 earlier with fever and started on IV fluids for suspected dehydration.  Mother comes out into the hallway frantically saying that her son does not look well and clinicians in the hallway (not the patient’s primary team) respond.  Expected team actions include a structured assessment to determine that the patient is in septic shock, enacting a plan to quickly deliver fluid resuscitation and broad spectrum antibiotics, and ensuring a shared mental model with a robust treatment plan. |
| **Primary Learning Objectives** | After this simulation, the participants will be able to:  1) Demonstrate knowledge and awareness of high-risk situations and errors leading to serious safety events in a pediatric hospital.  2) Apply communication techniques known to decrease errors in a high-risk situation. |
| **Critical Actions** | Assessment:  □ Check consciousness/ breathing/ color (PALS) or pediatric assessment  triangle  □ Primary assessment (ABCDE)  □ Obtain vital signs  □ Calculate Pediatric Early Warning Score (PEWS)- RN  □ Obtain SAMPLE History:  SIGNS/SX: Poor appetite, high HR  ALLERGIES: NKDA  MEDICATIONS: No home meds  PMH: None  LAST ORAL INTAKE: Sip of Gatorade 30 minutes ago (only PO today)  EVENTS: Limp x 1 week, fever x2 days, chills today, persistently febrile  since admission despite initiation of antibiotics; No UOP x 8 hours  □ Secondary assessment/ head-to-toe exam  □ Recycle BP q 5 minutes  □ Recognize bradypnea  Actions:  □ 100% FiO2 via non-rebreather  □ Places large bore IV x 2  □ Administers normal saline bolus with rapid technique (push-pull with 3-way  stopcock, pressure bag, or hand push small syringes) up to 60-100 mL/kg or  until normotensive  □ Orders broad spectrum antibiotics and make a plan to obtain and administer  them within an hour  □ Considers anti-pyretic (Tylenol/Motrin)  Clinical Reasoning:  □ Differential diagnosis of tachycardia  □ Recognize shock  □ Conduct watcher event with robust plan that creates a shared mental model  □ Consider calling rapid response team and primary team |
| **Learner Preparation or Prework** | All learners had completed 3 online Patient Safety Fundamentals modules prior to the simulation-based session. |

| Initial Presentation | | | |
| --- | --- | --- | --- |
| **Initial vital signs** | Temperature 39.4  HR 155  RR 22  BP 80/35  O2 Sat 95% on RA  PEWS 3 (for tachycardia; 3 in one category warrants watcher bedside huddle)  Weight 25 kg | | |
| **Overall Setting and Appearance** | Patient is a 6 year-old previously healthy boy now admitted for osteomyelitis and abscess with a newly placed PICC line for IV antibiotics. He now appears to be lethargic and Mom is at bedside concerned about his change in appearance. | | |
| **Confederates (e.g., standardized participants) and their roles in the room at case start** | Your 6 year-old son has been admitted to the hospital for the past 5 days receiving antibiotics for an infection. He suddenly looks less interactive and less responsive, so you rush out to the hallway to get help. Mother (played by facilitator) at bedside, concerned, stating “Johnny doesn’t seem to be waking up. He looks so sleepy. He wasn’t like this earlier. Can you help him please?”  One nurse in each session was assigned to be covering for the bedside nurse who was attending Code Response Training. The nurse was given a brief handoff on a laminated card with the following information about Johnny: This 6 year old boy was admitted 5 days ago for an extensive osteomyelitis and abscess and is here receiving IV antibiotics. He is being treated with IV Clindamycin through his PICC line. He was noted to be tachycardic to 130 earlier with fever and started on IV fluids for suspected dehydration.  The covering nurse and mother have information about Johnny and provide additional information when asked. It is expected that the covering nurse would give a summary when the team enters the room. | | |
| **HPI** | Significant Lab Values (provide only when asked):None  Significant Study Results (provide only when asked): None  Additional Information (provide only when asked):  Recent Events: None  Allergies: NKDA | | |
| **Past Medical/Surgical History** | **Medications** | **Allergies** | **Family History** |
| None | None | NKDA | None |
| **Physical Examination** | | | |
| **General** | Appears sleepy, PICC line in place | | |
| **HEENT** | Flushed cheeks | | |
| **Neck** | Normal exam | | |
| **Lungs** | Clear lungs, no retractions | | |
| **Cardiovascular** | Tachycardic with HR of 155, capillary refill 3-4 seconds | | |
| **Abdomen** | Normal exam | | |
| **Neurological** | Appears sleepy but initially arousable | | |
| **Skin** | Flushed cheeks | | |
| **GU** | Normal exam | | |

| Instructor Notes - Changes and CASE Branch Points | | |
| --- | --- | --- |
| **Intervention / Time point** | **Change in Case** | **Additional Information** |
| Mom appears in hallway from patient room yelling for someone to help her son. |  | “Johnny doesn’t seem to be waking up. He looks so sleepy. He wasn’t like this earlier. Can you help him please?” |
| Initial assessment- team enters room from hallway | Patient appears sleepy, but minimally responsive and intermittently moaning.  Initial VS:  T 39.4  HR 155  RR 22  BP 80/35  O2 Sat 95% on RA  PEWS 3 (HR) | Clinicians enter room, brief introductions and role assignment  The team is expected to apply a structured method for initial history (SAMPLE) and physical (ABCDE).  Covering nurse will share information on Johnny to team (per report, he had been started on fluids for HR of 130), now 155 and ill-appearing; Mom concerned  Mom: “He’s a healthy kid and is here for this bad infection. He just had a PICC line placed. I don’t understand what’s happening to him. Why is he so sleepy”?  The team is expected to complete an initial assessment, calculate a PEWS, note that the escalation algorithm requires a watcher event (bedside huddle), and call the primary team. |
| Reassessment | VS:  T 39.5  HR 154  RR 22  BP 75/30  O2 Sat 95% RA  PEWS 3  Unresponsive  Bounding peripheral pulses  Flash cap refill | The bedside nurse and physicians are expected to work together to convene a watcher event at the bedside. The team is expected to apply a structured assessment to determine that the patient is in septic shock, enact a plan to quickly deliver fluid resuscitation and broad spectrum antibiotics, and ensure a shared mental model with a robust plan. |

**Ideal Scenario Flow**

Clinicians in the hallway come to assist a mother who has come into the hallway shouting, “Please help my son. He’s really sleepy and doesn’t seem to be waking up”. The team immediately begins assessing the patient, obtaining vital signs, while one team member speaks with the mother to obtain more information. The team recognizes that the patient is tachycardic, febrile, and minimally responsive. Escalation can occur via a staff assist activation, rapid response call, or code blue activation, and the primary team should be called to the room. Roles should be identified, including a team leader. Supplemental oxygen is provided and an IV fluid bolus is ordered, ideally 20 cc/kg and administered rapidly using the push-pull technique. STAT antibiotics should be ordered and administered within one hour. The patient will likely be transferred to the Pediatric Intensive Care Unit for further fluid resuscitation or initiation of pressors and monitoring.

**Anticipated Management Mistakes**

1. Lack of shared mental model: Despite having completed the first scenario and debriefed the concept of shared mental model, a large number of teams did not share the mental model in this case. Attending physicians may have differed in their mental model based on their specialty, leading to confusion among team members on how to proceed with treatment. We found when teams correctly identified the shared mental model as septic shock and did so early, they quickly implemented interventions to help the patient (STAT antibiotics, rapid fluid bolus).
2. Confusion over acute care vs. critical care interventions: This scenario occurred in an acute care room with passersby responding to the emergency, including critical care teams. This led to confusion over supplies and equipment available on an acute care unit. For example, ICU teams were preparing to hang drips that may not be readily available in the acute care setting.
3. Knowledge deficit regarding rapid fluid administration (push-pull) vs. IV pump programming: We noticed that while many nurses were aware of push-pull technique for rapid fluid administration, many attending physicians did not, with the exception of ED and ICU providers. This prompted a brief discussion about the timeliness of administration with an IV pump compared with PALS guidelines and the need to be specific when ordering a fluid bolus for patients requiring a rapid fluid bolus. Additionally, we encouraged nurses to clarify whether push-pull was indicated, if not specified.
4. Lack of identified team leader: The majority of scenarios did not have an identified team leader. This made it challenging when there were multiple providers present, particularly when several may have been suggesting interventions. This was included as a debriefing question to highlight the importance of verbalizing who will be the leader to ensure role clarity.

Cue Card Information

Nursing Handoff:

This 6 year old boy was admitted 5 days ago for an extensive osteomyelitis and abscess and is here receiving IV antibiotics. He is being treated with IV Clindamycin through his PICC line. He was noted to be tachycardic to 130 earlier with fever and started on IV fluids for suspected dehydration.

Exam:

Cap refill 3-4 seconds

Clear lungs, no retractions

Responsive, normal neuro exam

Repeat Exam:

Unresponsive

Weak 1+ peripheral pulses

Cap refill 5-6 seconds
