# Supplementary material for: Code Response Training: Improving Interprofessional Communication
Source: MedEdPORTAL. 2021 May 19;17:11155. doi: 10.15766/mep_2374-8265.11155 (PMC8131416; doi:10.15766/mep_2374-8265.11155)
Supplement: Supplementary file 1 — Module 1 Patient Safety Fundamentals folderModule 2 Communication and Teamwork folderModule 3 Pulling It Together folderModule Instructions.docxFacilitators Guide.docxSimulation Case 1.docxSimulation Case 2.docxEquipment Checklist.docxObserver Checklist.docxDebriefing Guide.docxPostcourse Evaluation.docxShort-Term Follow-Up Activity.docxLong-Term Follow-Up Activity.docx [file mep_2374-8265.11155-s001.zip › H. Equipment Checklist.docx]

Code Response Training Equipment Checklist

- Curtains or screens to cover monitor and headwall in Simulation Center
- Tablets to run simulators
- AED (close by to bring shortly after it is requested)
- Code cart (close by to bring shortly after code is called)

| **Scenario #1- cafeteria** | **Scenario #2- acute care unit** |
| --- | --- |
| 1-3 yo HAL® with 3.5 plugged trach in place | 5 yo HAL® on stretcher |
| Trach bag- 3.0 trach ONLY, 3.5 trach missing | Leads & pulse ox ON, PICC line in L arm |
| Bedside table with 2 chairs on either side | IV pump & syringe pump on IV pole, D5 ½ NS infusing @65cc/hr |
| Chairs (2) | Patient monitor |
| IV with collection bag attached (once IV established) | IV attached with collection bag |
| Open bag of grapes on table | Code sheet at bedside |

Available equipment for Scenario 2:

- 10cc, 20cc, & 60 cc syringes
- IV start kits
- NS- 1 L bag
- IV tubing
- Stopcocks
- IV catheters- 22 & 24 g
- Ambu bag (2 sizes)
- Masks (3 sizes)
- Nonrebreather
- Nasal cannula
