# Supplementary material for: Code Response Training: Improving Interprofessional Communication
Source: MedEdPORTAL. 2021 May 19;17:11155. doi: 10.15766/mep_2374-8265.11155 (PMC8131416; doi:10.15766/mep_2374-8265.11155)
Supplement: Supplementary file 1 — Module 1 Patient Safety Fundamentals folderModule 2 Communication and Teamwork folderModule 3 Pulling It Together folderModule Instructions.docxFacilitators Guide.docxSimulation Case 1.docxSimulation Case 2.docxEquipment Checklist.docxObserver Checklist.docxDebriefing Guide.docxPostcourse Evaluation.docxShort-Term Follow-Up Activity.docxLong-Term Follow-Up Activity.docx [file mep_2374-8265.11155-s001.zip › I. Observer Checklist.docx]

**OBSERVER CHECKLIST**

Check the checkbox if this was addressed. Document your observations related to these specific areas (i.e. Nurse noted that breath sounds were normal 1 minute into the scenario. Bedside nurse delegated glucose check to teammate. Nurse and physician collaborated to compile SAMPLE history.)

**Checklist 1: Rapid Initial Assessment**

ABCDE Physical Exam

- Airway – Checks whether airway is patent.
- Breathing – Respiratory rate, breath sounds including air entry
- Circulation – HR, blood pressure, capillary refill, mental status, urine output
- Disability and Dextrose – Mini-neuro exam, blood sugar check if appropriate
- Exposure – Skin exam, temperature

Comments:

**Checklist #2: Communication**

- Establish a Shared Mental Model.
- Demonstrate respectful communication.
- Use closed loop communication.
- Did team members speak up and ask clarifying questions?
- Did everyone have a role at the bedside?

Comments:
