# Supplementary material for: Code Response Training: Improving Interprofessional Communication
Source: MedEdPORTAL. 2021 May 19;17:11155. doi: 10.15766/mep_2374-8265.11155 (PMC8131416; doi:10.15766/mep_2374-8265.11155)
Supplement: Supplementary file 1 — Module 1 Patient Safety Fundamentals folderModule 2 Communication and Teamwork folderModule 3 Pulling It Together folderModule Instructions.docxFacilitators Guide.docxSimulation Case 1.docxSimulation Case 2.docxEquipment Checklist.docxObserver Checklist.docxDebriefing Guide.docxPostcourse Evaluation.docxShort-Term Follow-Up Activity.docxLong-Term Follow-Up Activity.docx [file mep_2374-8265.11155-s001.zip › J. Debriefing Guide.docx]

Code Response Training Debriefing Guide

Scenario: Occluded Tracheostomy

- How did that go?
- What was the mental model for this patient? *(ask multiple people)*
- What if your mental model did not match other member of the group? How do you voice your thoughts/concerns? *(e.g. I thought trach plug – team thought child choking)*
- How do you call a code in the cafeteria? *(x#### on hospital phone; ###-###-#### from personal cell; no code blue buttons in cafeteria)*
- How long will the code team take to respond? *(a few minutes; can be delay if multiple code blues called at same time)*
- Where does the code cart come from? Who brings it? *(Central Supply on 1^st^ floor)*
- Is there an oxygen tank on the code cart? Is there suction on the code cart? *(No to both, but some parents may carry portable suction)*
- Are there tracheostomy replacement supplies on code cart? *(No; parents/ caregivers should carry emergency equipment with them at all times)*
- What equipment can you use on code cart for airway? *(2.5 ETT through stoma and bag or remove the tracheostomy tube, occlude stoma with gloved finger, and bag by mouth)*
- Where is the closest code cart? *(state location)*
- Where is the AED in the cafeteria? (*state* *2 nearest locations)*
- Did a team form? *When an ad hoc teams forms, it is important to have people who don’t know each other briefly introduce name, role and relevant skills.*
- Was there role clarity?
- Was a team leader designated?
- If a team leader does not step forward, ask who the team leader is.
- If your team does not have experience with tracheostomies, ask if someone does.

Everyone - Please share one take away from this simulation.

Scenario: Septic Shock

- How did that go?
- What was the mental model? *(ask a few people; e.g. septic shock)*
- How was the bolus given? *(ask team leader how quickly they expected it to run; run it on a pump over ½ hour or 1 hour? push-pull?)*
- Do you know how to do push-pull? *(Demonstrate- PALS algorithm calls for 20cc/kg of NS x 3 in first hour)*
- Can you give a bolus through PICC line? *(show them the PICC example & PIV catheter & explain that the PICC is longer which increases resistance)*
- Use a 20cc syringe for push-pull – (*25kg patient would get 25 syringes of normal saline)*
- How did you escalate this patient’s care – i.e. code blue or rapid response (RRT)? *(RRT comes in 15 minutes, while code is immediate; unresponsive, tachycardia, decreased BP)*
- Was there a clear team leader?
- How was teamwork?
- Were roles assigned?
- Was there a summary of what had been done so far?
- Were team members sharing what had been done? *(talking out loud)*
- Where are antibiotics kept on acute care units? *(antibiotics (ceftriaxone & vancomycin) are available in automated medication dispensing systems in Heme-Onc & ICUs, not available on acute care units and may take up to one hour to get a stat dose from pharmacy)*
- How can you expedite? Which antibiotic should be given first? *(ceftriaxone can go in over 5 minutes vs. 1 hour for vancomycin)*
- Oxygen – Patients in septic shock should be placed on a non-rebreather *(available in color drawers of code cart)*
- Ambu-bag cannot provide blow-by oxygen so do not place over the face unless you are bagging patient.

Everyone - Please share one take away from this simulation.
