# Supplementary material for: Code Response Training: Improving Interprofessional Communication
Source: MedEdPORTAL. 2021 May 19;17:11155. doi: 10.15766/mep_2374-8265.11155 (PMC8131416; doi:10.15766/mep_2374-8265.11155)
Supplement: Supplementary file 1 — Module 1 Patient Safety Fundamentals folderModule 2 Communication and Teamwork folderModule 3 Pulling It Together folderModule Instructions.docxFacilitators Guide.docxSimulation Case 1.docxSimulation Case 2.docxEquipment Checklist.docxObserver Checklist.docxDebriefing Guide.docxPostcourse Evaluation.docxShort-Term Follow-Up Activity.docxLong-Term Follow-Up Activity.docx [file mep_2374-8265.11155-s001.zip › K. Postcourse Evaluation.docx]

**EDUCATIONAL DESIGN EVALUATION**

**Program/Presentation Title:** **Interprofessional code response simulation training**

**Provider Unit #:**

**Date of Presentation:**

**Designate your role:**  Doctor Nurse NP PA RT

**DIRECTIONS: *Please complete this form using the rating scale by placing a check mark in the appropriate scale number box corresponding with evaluation statement/ question.***

**(1= low/poor to 5= high/excellent)**

| **How would you rate your overall achievement of each objective?** | **Low/**  **poor** | **Fair** | **Good** | **Very Good** | **High/**  **excellent** |
| --- | --- | --- | --- | --- | --- |
| Objective #1: Demonstrate knowledge and awareness of high-risk situations and errors leading to serious safety events in a pediatric hospital. |  |  |  |  |  |
| Objective #2: Apply communication techniques known to decrease errors in a high-risk situation. |  |  |  |  |  |
| Objective #3: Recall and incorporate effective techniques for teamwork and communication in your daily work. |  |  |  |  |  |
| **The faculty/presenters were effective.** | **Low/**  **poor** | **Fair** | **Good** | **Very Good** | **High/**  **excellent** |
| RN Facilitator |  |  |  |  |  |
| MD Facilitator |  |  |  |  |  |
| The application to and usefulness of the content in your practice. |  |  |  |  |  |
| The activity/session met your personal expectation. |  |  |  |  |  |
| **Did this session provide information that will cause you to change your practice?** | YES | | NO | | |
| If Yes, please describe new knowledge and/or skills you will apply in your practice: | | | | | |
| Which communication technique was new or reinforced in this simulation event? | | | | | |
| Which technique will you apply to your own and your team’s practice? | | | | | |
| The physical facilities were conducive for learning. |  |  |  |  |  |
| **Did you detect any conflict of interest, commercial support, product endorsement or unannounced off-label product use?** | | YES | | NO | |

What else would you need to learn to apply this to your practice? _________________________________________

_____________________________________________________________________________________________

Additional Comments: __________________________________**_________________________________________**

**_____________________________________________________________________________________________**
