# Supplementary material for: Code Response Training: Improving Interprofessional Communication
Source: MedEdPORTAL. 2021 May 19;17:11155. doi: 10.15766/mep_2374-8265.11155 (PMC8131416; doi:10.15766/mep_2374-8265.11155)
Supplement: Supplementary file 1 — Module 1 Patient Safety Fundamentals folderModule 2 Communication and Teamwork folderModule 3 Pulling It Together folderModule Instructions.docxFacilitators Guide.docxSimulation Case 1.docxSimulation Case 2.docxEquipment Checklist.docxObserver Checklist.docxDebriefing Guide.docxPostcourse Evaluation.docxShort-Term Follow-Up Activity.docxLong-Term Follow-Up Activity.docx [file mep_2374-8265.11155-s001.zip › M. Long Term Follow-up Activity.docx]

**Code Response Training Long Term Follow up Activity**

**(4-6 months post course)**

1. Match the following statements to the appropriate safety techniques:

a. Code response participant to the 2^nd^ responder: “Go call a code”.

2^nd^ Responder replies, “I just called a code”.

*Technique-Closed loop communication*

b. Nurse after the code response: “I thought we needed to change the trach, but I was waiting

for the doctor to tell me”.

*Technique- Speaking up*

c. Nurse in the code response: “I know how to change a trach.”

Physician in code response: “Great. Go ahead. Tell me what to do and I’ll assist.”

*Technique- Deference to expertise*

d. When responding to an event, the first thing you want to do when you arrive.

*Introductions*

Answer the following True/False Questions:

1. Standardized communication is useful during times of stress or when you must deliver important patient or operational information.

*True. Standardized communication is an effective way to communicate during times of stress. It helps in getting the immediate attention of the listener and helps convey a shared mental model.*

1. Situational awareness is the state of knowing and understanding all the small details of a situation, whether or not they are relevant.

*False. Situational awareness is the state of knowing and understanding what is happening inside your immediate environment, the “big picture”.*

1. Assertiveness is the ability to know when to speak your mind openly depending on who is present.

*False. Assertiveness is the ability to speak openly regardless of who is present.*

1. What were you able to take away from the class and apply to your clinical practice?
2. What else would you need to learn to apply this to your practice?
3. Additional comments:
